# The readiness–preparedness bias: recalibrating monitoring logic

**DOI:** 10.3389/fphys.2026.1855985

**Published:** 2026-06-01

**Authors:** Karol Kruczek, André Rebelo, Tim Gabbett, Michał Nowak

**Affiliations:** 1 Sports Science Department, Next Generation Performance, Kraków, Poland; 2 CIDEFES, Research Center in Sport, Physical Education, and Exercise and Health, Lusófona University, Lisbon, Portugal; 3 Faculty of Sport, Centre for Research, Education, Innovation, and Intervention in Sport (CIFI2D), University of Porto, Porto, Portugal; 4 COD, Center of Sports Optimization, Sporting Clube de Portugal, Lisbon, Portugal; 5 Gabbett Performance Solutions, Brisbane, QLD, Australia; 6 Department of Physical Culture Sciences, Collegium Medicum named after Doctor. Władysław Biegański, Jan Długosz University in Częstochowa, Częstochowa, Poland

**Keywords:** load management, athlete assessment, external load, decision-making, normative benchmarks, sport-specific capacity, performance profiling, return to sport

## Abstract

This perspective argues that readiness monitoring metrics may be given disproportionate decision-weight relative to sport-specific preparedness indicators when daily load choices are made for athletes whose chronic physical capacity remains below the demands of competition. We term this tendency the “Readiness–Preparedness Bias” and propose a theoretical model in which readiness monitoring may assume greater practical importance as athletes approach relevant preparedness standards, while still retaining supportive value in underprepared athletes by helping practitioners calibrate progressive exposure and identify clinically meaningful deviations. We synthesise evidence from athlete monitoring, training theory, normative profiling, and return-to-sport literature to highlight that monitoring data are most useful when interpreted against measurement error, contextual dependence, and current sport demands. We also highlight the cognitive, organisational, and interpretive costs of dense monitoring systems. Our aim is not to reject monitoring, but to recalibrate its role: in underprepared athletes, monitoring should primarily guide progressive exposure and dose prescriptions, thereby supporting long-term physical development, sport-specific adaptation, and the gradual accumulation of the capacities required for higher-level performance, rather than repeatedly diluting training stimuli in response to trivial short-term fluctuations.

## Introduction

High-performance sport has been reshaped by the data revolution, shifting coaching from primarily intuition-based practice toward more evidence-informed decision-making (Coutts, 2014; Morgulev et al., 2018). Practitioners now use a broad range of monitoring technologies to quantify external and internal load, and elite teams report using these data to adjust daily training prescriptions (Buchheit et al., 2021).

However, in some settings, the growing volume of monitoring data may shift decision-making priorities toward managing short-term fluctuations in acute status rather than progressively developing long-term physical capacity (Jeffries et al., 2022). An important question in contemporary strength and conditioning, and increasingly in clinical rehabilitation, is whether advanced monitoring and day-to-day load adjustment strategies are being applied in populations whose chronic physical preparation has not yet reached the level at which such approaches provide valuable insights.

In this perspective, we define the “Readiness–Preparedness Bias” as the systematic overweighting of acute readiness indicators (e.g., neuromuscular test performance) relative to preparedness determinants (e.g., benchmarked physical capacities) when making daily load-management decisions, particularly in athletes who have not yet reached adequate physical preparation. Importantly, at this stage we use the term bias as a theoretical and heuristic descriptor of a proposed decision tendency, rather than as evidence of an empirically settled and universally demonstrated error pattern across sporting contexts. In some settings, single metrics or otherwise limited monitoring indicators are used to inform important training or competition decisions, despite the absence of any single definitive marker of athlete status and the uncertain validity or clinical applicability of several commonly used measures (Halson, 2014; Duignan et al., 2020; Jeffries et al., 2020). Accordingly, the practical utility of readiness data should be viewed as conditional rather than universal. The same monitoring-derived response may have markedly different implications depending on athlete health status, competitive level, environmental context, and prevailing cultural beliefs.

**Figure 1:**
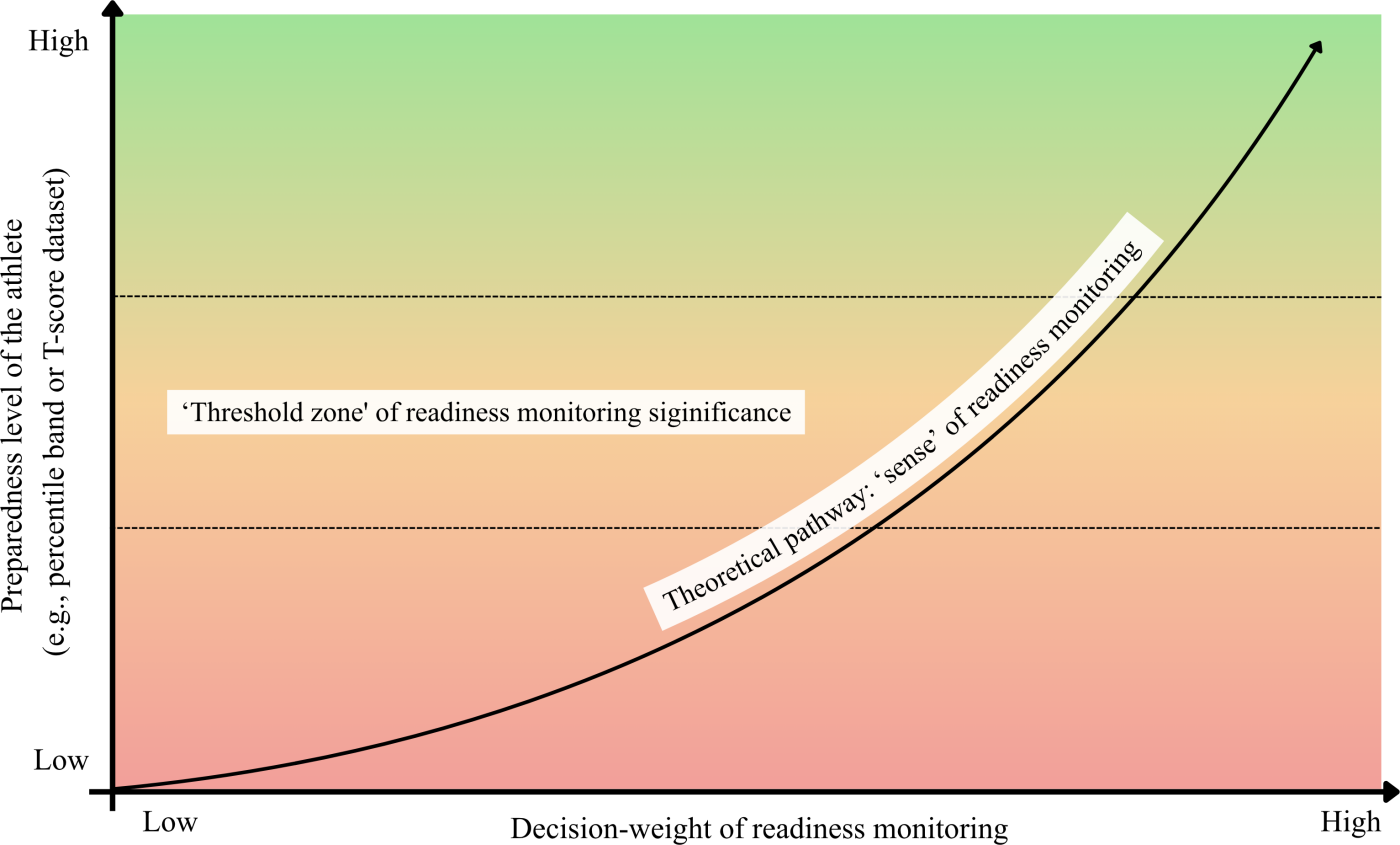
A conceptual model of the decision-weight assigned to readiness monitoring as a function of proximity to sport-specific normative benchmarks.

This article is not an argument against monitoring *per se*, but against assigning disproportionate priority to acute readiness metrics in some contexts. Monitoring is most valuable when it calibrates dose without suppressing the intended developmental stimulus unless deviations are meaningfully outside measurement error (Jeffries et al., 2022; Impellizzeri et al., 2023). Importantly, this does not imply that readiness monitoring lacks value in underprepared athletes; rather, its primary role in such contexts may be to calibrate progressive exposure, detect meaningful variations from expected tolerance, and support communication. We therefore propose a theoretical model in which the decision-weight assigned to acute readiness variables is conceptualised as a function of the athlete’s objectively benchmarked physical preparedness, referenced against normative values derived from the same or a closely comparable sporting population (**Figure 1**). Because direct experimental or prospective comparisons between readiness-led and preparedness-weighted decision logics remain lacking, this perspective should be read as a testable hypothesis and future research agenda.

## Current understanding of readiness and preparedness

Within the present framework, preparedness refers to the chronic, sport-specific capacity to tolerate, recover from, and adapt to the exposures imposed by training and competition. It is the accumulated product of long-term structural, neuromuscular, metabolic, and coordinative adaptations and therefore reflects the athlete’s underlying ability to repeatedly express performance-relevant qualities over time, rather than a momentary state of *freshness* (Gabbett, 2016; Jeffries et al., 2022; Boullosa et al., 2023). By contrast, readiness refers to the athlete’s acute state of functional availability to train or compete on a given day—that is, the extent to which this underlying capacity can be expressed under the transient influence of fatigue, recovery status, soreness, psychological state, and other short-term modulators (Saw et al., 2016; Kellmann et al., 2018; Beato et al., 2024). Within a multidimensional monitoring framework, readiness is not a standalone endpoint, but a contextual indicator of current state and short-term training effects. Its value depends on longitudinal trends, recent load exposure, and the athlete’s position relative to individual baselines and sport demands. Likewise, preparedness should not be inferred from normative scores alone, but interpreted alongside athlete-specific baselines and prior training history. Both constructs are intentionally broad rather than purely physiological, as preparedness and readiness are shaped by biopsychosocial, economic, institutional, and sport-specific contextual factors that may constrain how physical capacity is developed, expressed, and interpreted.

## Moving normative maps and objective performance indicators

To operationalise preparedness, practitioners must recognise that the physiological demands of elite competition are a moving target, meaning older performance profiles may no longer reflect contemporary demands (Barnes et al., 2014; Gabbett, 2020; Woodhouse et al., 2021; Lago-Peñas et al., 2023). This creates a calibration problem in which athletes are benchmarked against outdated datasets, highlighting the need for dynamic normative maps that capture the multidimensional evolution of sport.

Recent data from elite football illustrate this point. English Premier League match demands continue to evolve, with increases in total distance, high-intensity distance, high-speed running, and sprint distance across the last decade (Allen et al., 2025). Team success also appears to relate more closely to contextualised high-intensity outputs than to undifferentiated running volume alone (Mandorino et al., 2025; Allen et al., 2026). If readiness monitoring is used mainly to preserve lower-level outputs without reference to actual normative benchmarks, training decisions may become anchored to capacities below the target competitive standard. For example, in elite men’s football, maximal running-speed benchmarks vary by league and position, typically falling around 30–32 km/h and reaching 35–37 km/h in the fastest match actions (Oliva-Lozano et al., 2020, Oliva-Lozano et al., 2023; Altmann et al., 2023, Altmann et al., 2026). If an athlete’s sprint capacity remains materially below these standards, routine deloading in response to trivial day-to-day fluctuations is unlikely to close a meaningful preparedness deficit. In such cases, planned progression of maximal-speed exposure may be required, with monitoring used primarily to calibrate dose rather than attenuate the developmental stimulus, unless more credible contextual signs (e.g. injury, illness, or poor mental state) justify load modification.

The same logic applies beyond football. The UFC Performance Institute (2018; UFC Performance Institute, 2021), although best treated as supplementary grey literature, show how sport-specific profiling can move beyond generic fitness toward weight-class-specific benchmarks for force, power, and metabolic outputs. Their successive editions suggest that normative standards themselves can rise over short time spans. Current systematic review evidence establishes a provisional physiological profile associated with higher competitive levels, demonstrating that elite competitive outcomes in mixed martial arts are strongly differentiated by superior relative lower-body force and power output, alongside robust alactic anaerobic capacities; accordingly, selected proxy metrics derived from simple performance tests may provide indirect but practically useful signals of adequate sport-specific preparedness when interpreted cautiously (James et al., 2016).

Recent normative datasets increasingly move beyond single output metrics toward multidimensional profiling frameworks that often reflect not only performance outcomes but also the movement strategies underlying them, as illustrated by recent work in rugby league and basketball (McMahon et al., 2022a, McMahon et al., 2022b; Berberet et al., 2024; Suárez-Balsera et al., 2025). This approach is equally relevant in many court sports, where force-plate-derived, phase-specific braking characteristics from vertical jump testing may distinguish meaningful differences in competitive level, playing status, and selected sport-relevant capacities more sensitively than traditional jump outcomes (Geneau et al., 2023; Philipp et al., 2023; Spyrou et al., 2024; Radovic et al., 2024; Cabarkapa et al., 2024, Cabarkapa et al., 2025).

The practical implication is straightforward: readiness status interpreted without benchmark context may offer only limited descriptive value, whereas preparedness should be judged against sport-, level-, sex-, role-, and task-specific reference criteria selected for their causal proximity to competitive demands.

## Methodological risks in decision-making process

An important problem in monitoring-based decision-making is that many acute changes are interpreted as meaningful despite the limited reliability, context dependence, and measurement precision of the tools used. In academy football, commonly utilised countermovement jump (CMJ) measures have demonstrated minimal detectable change thresholds in the range of approximately 14.6–23.7%, while several isometric force-time variables exhibit even poorer practical utility due to critically elevated measurement error (Springham et al., 2024). Anicic et al. (2023) showed that the availability of numerous CMJ-derived variables does not justify their indiscriminate use, as only 24 of 45 metrics demonstrated acceptable reliability. Furthermore, a contemporary scoping review of force plate monitoring literature identified substantial methodological heterogeneity in test selection, metric definition, calculation procedures, and data-collection protocols (Badby et al., 2025). Additionally, inappropriate reporting of monitoring-session results may add unnecessary random variation to the assessment process, while daily averaging across repeated trials appears to be the preferable approach for enhancing the signal-to-noise ratio (Kennedy and Drake, 2021). Further, selected markers derived from fatigue monitoring should not be assumed to directly reflect progression in sport performance, since short-term reductions in neuromuscular outputs may occur alongside meaningful improvements in physical capacities (Roe et al., 2016). Borresen and Lambert (2009) highlighted that the search for a single marker capable of simultaneously quantifying fitness, fatigue, and performance is fundamentally problematic, while training–performance models remain constrained by poor accuracy and the highly individual nature of training responses.

Importantly, readiness must not be conceptualised as a unitary latent state adequately captured by a single dashboard metric. Systematic review evidence indicates that subjective and objective markers of athlete well-being often fail to correlate, and that subjective measures may demonstrate superior sensitivity to both acute and chronic load perturbations (Saw et al., 2016). When athlete monitoring becomes a burdensome and poorly contextualised administrative exercise, the resulting data may be less informative than assumed, as opaque systems with limited feedback can compromise reporting honesty and data accuracy at the point of collection (Coventry et al., 2023). Therefore, when practitioners react to small, poorly contextualised fluctuations without considering measurement error or recent exposure, they may over-interpret normal biological variation and unnecessarily dilute the intended training stimulus, crucially mistaking necessary homeostatic/allostatic regulation for maladaptation (Kiely, 2018; Coutts, 2014).

## Load management and programming considerations

These problems have direct programming implications. Without a sound theoretical framework, load measurement becomes descriptive rather than prescriptive (Impellizzeri et al., 2019). A more rigorous solution is to rank monitoring variables according to their causal proximity to the adaptation sought within a given training block (Impellizzeri et al., 2023). Accordingly, metrics closest to the target adaptation should carry the greatest decision weight, whereas more distal moderators should contextualise, rather than dominate, training decisions unless deviations are clearly meaningful, thereby shifting the focus of load management from acute optimisation toward long-term physical robustness (Kalkhoven et al., 2021). Thus, when for example foundational strength remains insufficient, prioritising advanced or predominantly power-oriented methods may divert training away from the more pressing need for basic physical development. This is particularly consequential because maximal strength underpins force–time characteristics and provides the basis for rapid force production and muscular power expression (Cormie et al., 2011; Suchomel et al., 2016, Suchomel et al., 2018).

This same logic applies to injury-related decisions, where the temptation to overinterpret isolated readiness markers may be especially strong. The contemporary literature likewise cautions against reductionist thinking, emphasizing that injury emerges from multiple interacting determinants and cannot be reliably predicted by any isolated functional test (Bittencourt et al., 2016; Bahr, 2016). Because injury prediction from isolated screening tests is currently unreliable, readiness assessments should be framed as contextual inputs for load prescription rather than binary *safe/unsafe* indicators. Consequently, an exclusive focus on preserving short-term *freshness* may constrain the repeated exposure needed to support long-term robustness (Gabbett, 2016). Greater preseason participation has been associated with lower in-season injury risk, supporting the premise that building chronic exposure can enhance athlete robustness within a broader workload–injury aetiology framework (Windt and Gabbett, 2017; Windt et al., 2017). When descriptive monitoring data are used to systematically reduce training stimuli, practitioners risk promoting biological *fragility* rather than positive adaptation (Gabbett, 2016; Kiely, 2018).

From this perspective, load management should be embedded within a pre-planned yet responsive periodisation framework, not as a reactive brake on it. In elite sport, this need not imply a rigid block model: contemporary planning more often requires the parallel development of multiple motor qualities, the maintenance of athlete availability for demanding physical efforts, and strategically planned variation with only bounded daily adjustments in response to athlete status (Kiely, 2012; Mujika et al., 2018; Berryman et al., 2019; Gabbett, 2016). Responsiveness to biological data is therefore not synonymous with systemic de-loading; instead, feedback should be used to tactically redistribute stressors while preserving progression toward a minimum effective training dose. This distinction is particularly important in injured athletes, in whom global preparedness for repeated competition may remain low, while readiness monitoring may still be needed to determine whether the involved tissue can tolerate the intended local training stress (Gabbett et al., 2021; Gabbett and Oetter, 2025). Instead of blunt reductions driven by minor readiness fluctuations, practitioners should strategically manipulate stress–recovery dynamics across the microcycle and progressively expose athletes to loads that enhance tissue-specific capacity (Impellizzeri et al., 2020a, Impellizzeri et al., 2020b; Gabbett, 2020; Sousa et al., 2024). In hypertrophy-oriented training, variability should be planned rather than reactive, as modest variation may support hypertrophic adaptation, whereas excessive high-frequency changes may dilute the hypertrophic stimulus (Kassiano et al., 2022). A highly reactive interpretation of day-to-day fluctuations sits uneasily with historical fundamental training models that treat transient fatigue as a normal component of adaptation (Banister et al., 1975; Matveev, 1981) rather than as a signal that training stress should be routinely reduced. Borszcz et al. (2022) suggest that the appearance of acute fatigue in monitoring data should not automatically be interpreted as a negative outcome, as a transient, non-impairing fatigue state may accompany the adaptive responses to concentrated training stress.

A similar concern applies to durability. Although the construct originates mainly from endurance sport, its underlying logic may be heuristically useful in team sport and rehabilitation: athletes must sustain technical, mechanical, and psychological function under fatigue (Maunder et al., 2021; Spragg et al., 2023). By repeatedly diluting the training stimulus in response to trivial readiness fluctuations, practitioners may deprive athletes of the very exposure required to build resilience to elite competition.

In practical terms, training programming should follow a simple decision hierarchy: (1) identify the primary performance or adaptation target; (2) determine which physical qualities matter most in the current block; (3) interpret readiness metrics against individual variability, recent context, and normative preparedness guidelines; (4) meaningfully modify load only when observed changes are likely to be real and decision-relevant; and (5) where appropriate, prefer redistribution of stressors over automatic suppression of total stimulus.

## Psychological and behavioural mechanisms

Several cognitive mechanisms may help explain how this *bias* emerges in applied settings, although direct evidence within athlete-monitoring environments remains limited. When every acute decrement in neuromuscular performance is framed as a warning signal, athletes may begin to interpret normal training-related fluctuations as indicators of susceptibility rather than as expected features of adaptation. Excessive monitoring may also foster expectancy-related nocebo responses when negatively framed readiness feedback is repeated without adequate context; systematic reviews indicate that placebo and nocebo effects can meaningfully influence sport performance (Hurst et al., 2020; Chhabra and Szabo, 2024).

Practitioners may be as vulnerable to bias as the athletes they monitor. Through Kahneman’s dual-process lens, high-pressure environments may favour fast, intuitive and more emotional “System 1” responses to salient daily metrics, at the expense of the slower “System 2” reasoning needed to sustain long-term periodisation goals (Kahneman, 2011). This more strategic and reflective thinking mode may support delayed gratification and the prioritisation of chronic adaptation over immediate *freshness* (Metcalfe and Mischel, 1999). Recent work suggests that increasing data volume may complicate rather than simplify decision-making when interpretation is heuristic, poorly structured, or weakly integrated with prior probabilities and contextual knowledge (Yung et al., 2024), while confirmation bias may further amplify fatigue-related concerns and downplay evidence of adaptation capacity and load tolerance (Beato et al., 2025).

## The monitoring tax

Athlete monitoring is not a neutral act of observation. It carries physiological, financial, cognitive, organisational, and interpretive costs, and its value depends less on the volume of data collected than on whether those data improve decisions beyond what could be achieved through simpler observation (West et al., 2021). Dense monitoring systems may reduce decision clarity when staff must interpret multiple, only partially convergent signals under time pressure, while burdensome or poorly explained procedures may also compromise athlete engagement and data quality (Saw et al., 2016; Neupert et al., 2022; Coventry et al., 2023; Yung et al., 2024). The key question is therefore not whether more data can be collected, but whether an additional monitoring layer provides positive net decision value—especially in athletes whose primary developmental need is still the accumulation of training exposure rather than more precise differentiation of day-to-day readiness (Impellizzeri et al., 2023).

## Operationalising and empirically testing the sport-specific preparedness benchmark

To make the proposed model practically usable, practitioners need a clearer way to distinguish which qualities define the main performance gap and which variables mainly modulate their short-term expression. To operationalise this hierarchy, it may be useful to distinguish between performance determinants and moderating factors. Determinants are the qualities that must be enhanced to improve the target outcome, whereas moderating factors influence the expression of performance without defining the primary performance gap (Washif and Pyne, 2026). In the present framework, chronic preparedness is more closely reflected by determinant capacities, while many day-to-day readiness variables function chiefly as moderators. The practical mistake arises when variables of mainly moderating value are allowed to override the strategic development of underpowered determinant qualities. Once a motor quality is no longer a meaningful bottleneck, continued increases in training dose are likely to produce diminishing returns, which shifts priority toward less-developed capacities (Vial et al., 2025; Pelland et al., 2026).

**Figure 2:**
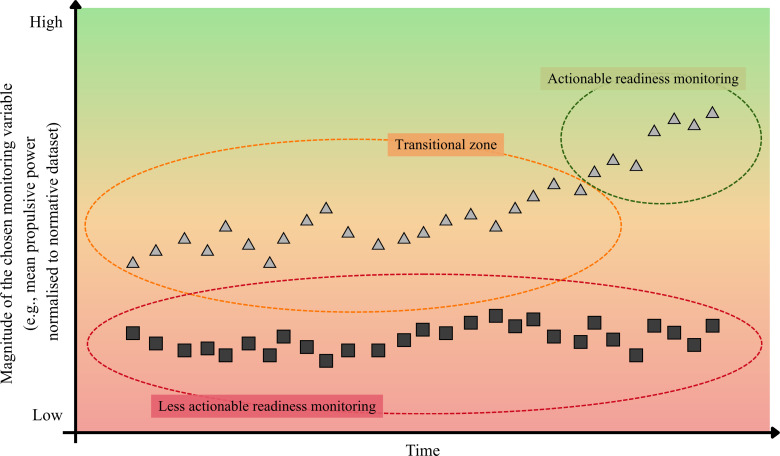
Conceptual illustration of two athletes’ monitoring trajectories relative to normative reference data and the resulting practical utility of readiness monitoring for decision-making. Triangles and squares represent serial monitoring outcomes (e.g., daily mean scores) for Athlete A and Athlete B, respectively, plotted over time against a normative reference continuum. The lower zone (“less actionable readiness monitoring”) denotes a level of physical preparedness at which day-to-day monitoring outputs have limited practical value for guiding staff decision-making, as the athlete’s underlying training status remains insufficient for acute fluctuations to be interpreted as meaningful determinants of load adjustment. The middle zone (“transitional zone”) reflects a stage at which monitoring outputs begin to acquire greater interpretive and practical value as the athlete approaches relevant sport-specific preparedness standards. The upper zone (“actionable readiness monitoring”) represents a level of preparedness at which readiness monitoring becomes more decision-relevant, such that short-term fluctuations may more appropriately inform load-management decisions. This figure is theoretical and intended to illustrate the proposed relationship between an athlete’s preparedness level, their position relative to normative data, and the applied utility of readiness monitoring.

The sport-specific preparedness benchmark is therefore better conceptualised as a gradient or zone than as a single strict threshold. Importantly, this gradient refers to the relative influence of readiness metrics on decision-making, not to their absolute usefulness, meaning that readiness remains informative below a given preparedness level but should carry less weight until a more sufficient foundation is in place. The decision-weight of readiness scores should increase non-linearly as the athlete approaches the relevant preparedness standard (**Figure 1**). This principle is illustrated in **Figure 2** using an example CMJ-derived mechanical metric for Athletes A and B, where the athletes’ serial results and overall trajectory relative to the normative preparedness continuum determine the decision-weight assigned to this parameter when considering potential modifications to training prescription. Depending on context, that standard may be expressed as a percentile band, a T-score, a minimum physical standard, or a return-to-sport criterion. Central tendency may be appropriate when the objective is to establish a broad developmental threshold in heterogeneous populations, whereas upper-quartile standards may better reflect the chronic capacity expected in high-performance settings. However, it should be remembered that the limited availability of elite-level normative data in the literature remains a key constraint (Hughes et al., 2001). Rather than replacing existing monitoring frameworks, the present hypothesis should be operationalised by extending established good-practice approaches, such as the seven-step framework proposed by Rebelo et al. (2026), to include explicit contextualisation of selected readiness metrics against available normative datasets and group-specific reference values. .

To move this scheme beyond theoretical plausibility, a major research priority is to test whether weighting readiness metrics according to proximity to normative benchmarks improves training decisions, competitive performance, or return-to-sport outcomes. This could be addressed through sequential mixed-methods designs, beginning with qualitative case mapping to identify how practitioners currently balance readiness and preparedness across different sport contexts, followed by prospective quantitative comparisons between standard readiness-led monitoring and preparedness-weighted decision-support models. Such studies should deliberately sample contrasting cases, including team, individual, and clinical settings, to examine when the proposed hierarchy is useful, context-dependent, or insufficient.

## Conclusion

This perspective does not reject athlete monitoring; it argues for a different decision hierarchy. Acute readiness metrics should exert their greatest influence mainly once the athlete is sufficiently prepared relative to the objectively measured demands of the sport. Before that point, the dominant task is to build chronic capacity through progressive loading, guided—but not governed—by monitoring data. Furthermore, it is the authors’ contention that athletes within early developmental or rehabilitative stages—where the priority is physiological growth rather than short-term performance optimisation—should be managed primarily via incremental exposure and planned load distribution (with bounded day-to-day adjustments), prioritising the accumulation of determinant capacities required for competition. The presented claim is testable in principle, and future work should examine when, for whom, and under what constraints readiness monitoring truly adds net decision value.

## Data Availability

The original contributions presented in the study are included in the article/supplementary material. Further inquiries can be directed to the corresponding author.
